# Site-specific fluorescence double-labeling of proteins and analysis of structural changes in solution by Fluorescence Resonance Energy Transfer (FRET)

**DOI:** 10.1016/j.mex.2018.03.006

**Published:** 2018-03-31

**Authors:** Meera J. Patel, Gulden Yilmaz, Lavesh Bhatia, Esther E. Biswas-Fiss, Subhasis B. Biswas

**Affiliations:** a Program in Biotechnology, Department of Medical Laboratory Sciences, College of Health Sciences, University of Delaware, Newark, DE 19716, United States; b Department of Molecular Biology, Rowan University, Stratford, NJ 08084, United States

**Keywords:** Site-specific fluorescence labeling of proteins and analysis of structural changes in solution by Fluorescence Resonance Energy Transfer (FRET), FRET analysis, FlAsH, Alexa_568_ maleimide, steady-state photon counting spectrofluorometer, Site-specific labeling, intramolecular distance determination, conformational changes

## Abstract

Fluorescence Resonance Energy Transfer (FRET) is a well-known methodology for detection and quantitation of structural changes of proteins in solution. FRET requires site-specific protein labeling with two fluorophores, one of which functions as an energy donor and the other one as an energy acceptor. However, the site-specific labeling of protein is often complex and difficult, particularly when inserting two fluorophores in specific sites. We have examined several protein labeling approaches with a varying degree of success. Described here is a dual labeling strategy that worked reproducibly in a number of protein targets and we believe will be applicable to a variety of proteins, which have few or no native cysteine (Cys) residues. We have successfully double-labeled DnaA protein of *Bacillus anthracis*, which lacks intrinsic Cys residues. A cysteine residue was inserted at the N-terminus by *in vitro* mutagenesis and a Cys-Cys-Phe-Gly-Cys-Cys (CCPGCC) sequence at the C-terminus by PCR. This protein was labeled site-specifically with a fluorescein derivative, FlAsH, at the CCPGCC sequence followed by Alexa_568_ maleimide at the N-terminus Cys residue. Structural changes of the protein with nucleotide, DNA and an inhibitor protein binding were determined by FRET analysis of the double-labeled protein. This comprehensive novel methodology for site-specific protein labeling with different fluorophores is applicable for understanding different *in vitro* proteomic structural studies. Here, we describe a verified technique used for FRET spectral analysis and quantitative evaluation of structural changes using fluorophore labeled DnaA protein constructs as an example.

## Highlights

• Insertion of a Cys4 sequence and single Cys motif allowedsite-specific labeling with FlAsH and Alexa_568_ of a protein.
• FRET is a well-known technique useful for analysis of structural changes of proteins labeled with donor and accepter fluorophores.
• Inter-fluorophore distances and proximity ratios of the peakintensities are used to understand structural changes.

## Graphical abstract

**Figure:**
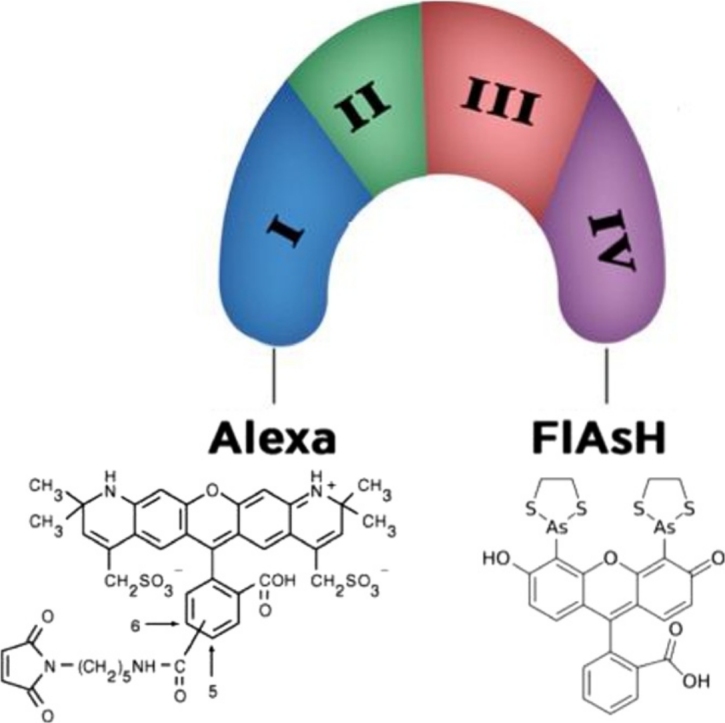

**Specifications Table**

**Table:** 

Subject area	*Biochemistry and Biophysics*
More specific subject area	*Fluorescence spectroscopy of fluorophore labeled proteins*
Method name	Site-Specific Fluorescence Labeling of Proteins and Analysis of Structural Changes in Solution by Fluorescence Resonance Energy Transfer (FRET)
Name and reference of original method	• Adams, Stephen R; Tsien, Roger Y, Preparation of the membrane-permeant biarsenicals FlAsH-EDT2 and ReAsH-EDT2 for fluorescent labeling of tetracysteine-tagged proteins, Nature Protocols, 3 (9) (2008) 1527–34.
	• S. Granier, S. Kim, A.M. Shafer, V.R. Ratnala, J.J. Fung, R.N. Zare, B. Kobilka, Structure and conformational changes in the C-terminal domain of the beta2-adrenoceptor: insights from fluorescence resonance energy transfer studies, J Biol Chem, 282 (2007) 13895-13905.
	• J. Lakowicz, Principles of Fluorescence Spectroscopy, 3 ed., Springer US, 2006.
Resource availability	• http://www.horiba.com/scientific/products/fluorescence-spectroscopy/software/fluoressence/fluoressence-837/
	• http://www.fluortools.com/software/ae

## Method details

### Background

The purpose of this study was to devise an optimal procedure to quantify the structural changes of a protein occurring in solution. Although there are various techniques that serve as means to measure conformational changes, we have chosen Fluorescence Resonance Energy Transfer (FRET) as the methodology because of its application to proteins *in vitro* and *in vivo*. FRET allows researchers to look at intermolecular and intramolecular interactions. FRET is a profound and verified approach, initially described by Theodor Förster, for detection of structural changes of a protein [1]. Although FRET is a straightforward approach, many factors are involved in assuring the accuracy of this procedure.

First, site-specific labeling of the target protein must be carried out in a manner to ensure that the correct amino acids or motifs are labeled. Without site-specific labeling, fluorophores can label the protein non-specifically invalidating the structural analysis. In this study, fluorescin arsenical hairpin binder-ethanedithiol (FlAsH-EDT_2_) fluorophore specifically crosslinks to a tetracysteine CCPGCC motif, whereas, the Alexa_568_ maleimide crosslinks to the sulfhydryl group of a Cys residue, introduced by site-specific mutagenesis, similar to that described earlier by Granier et al. [2]. Second, the chosen fluorophores must be within a distance of ∼10 nm for spectral overlap to allow appreciable transfer of energy from the donor to acceptor [3]. FlAsH, invented by Roger Tsien, is an organo-arsenic derivative of fluorescein and used for labeling a specific α-helical CCPGCC sequence in proteins [4]. It is important that the protein sequence does not contain a high number of cysteines as FlAsH and Alexa_568_ may bind non-specifically to these cysteines. Shih et al. conducted a FRET based intermolecular experiment between two myosin chains to assess the prestroke conformational changes [5]. In order to measure the structural change occurring upon nucleotide binding, native cysteines were removed from the myosin-II and a new cysteine residue was inserted in a specific location to label with donor or acceptor dyes. Shih and his group labeled two separate polypeptides with distinct donor and acceptor fluorophores and then measured the protein-protein interactions of the complex [5]. However, in order to study the intramolecular interaction both fluorophore probes must be placed on the same polypeptide. Another important feature of FlAsH is that it only fluoresces when cross-linked to a CCPGCC sequence. In addition, a wide range of Alexa Fluor dyes are commercially available from Life Technologies (part of Thermo Fisher Scientific) that excite at different wavelengths and form suitable acceptor fluorophores. Following earlier reports, we chose Alexa_568_ as the acceptor fluorophore in our study [2], assuring that the excitation and emission wavelengths for FlAsH and Alexa_568_ fluorophores overlap ideally to allow maximal energy transfer. In order to accurately calculate the distance between the fluorophores and to quantify the energy transfer occurring within the protein, the raw data needed to be normalized with a uniform procedure throughout the study. We have used a simple methodology to calculate the crucial values that demonstrate conformational changes for DnaA protein of *Bacillus anthracis* and the strategy is applicable to other proteins as well.

This method can also be applied to proteins with a number of native cysteines. The Cys residue(s) must be removed by *in vitro* mutagenesis to Ala or Ser before introduction of additional Cys and CCPGCC motif as shown by Granier et al. [2]. Needless to mention, the FRET study is not limited to the fluorophores discussed here. Life Technologies (Carlsbad, CA) provides a wide variety of commercial fluorescent probes. Here, we have presented with a straightforward and quick procedure allowing us to conduct intramolecular and intermolecular studies using FRET with available molecular tools.

### Experimental procedures

#### Oligonucleotides, reagents, DNA sequencing and fluorophores

All buffers and solutions used in this study were prepared using ACS reagent grade chemicals from Fischer Scientific (Pittsburgh, PA). The modified oligonucleotides used to construct the wild-type DnaA_BA_ (DnaA_wt_) and DnaA_1_ mutant by PCR amplification and *in vitro* mutagenesis, were synthesized by Sigma-Genosys Corp. (Woodlands, TX). The procedure for cloning the DnaA_BA_ from *Bacillus anthracis* (Stern strain) has been discussed previously [6]. We chose Alexa_568_ maleimide (Life Technologies, Grand Island, NY) to label a single cysteine in the protein as an acceptor fluorophore and FlAsH-EDT2 (Life Technologies, Grand Island, NY) to label the specific CCPGCC sequence.

##### Buffers

The purified DnaA_wt_ and DnaA_1_ mutant were stored in a buffer consisting of 10% glycerol, 20 mM Hepes (pH 7.5), 1 mM EDTA (pH 8.0), 5 mM MgCl_2_, 0.01% NP40 and 0.5 mM TCEP and frozen in −80 °C until labeling. The buffer used for protein recovery after labeling contained 10% glycerol, 20 mM Hepes (pH 7.5), 5 mM MgCl_2_, 0.01% NP40 and 0.5 mM TCEP. Proteins were added to anisotropy buffer, containing 20 mM Hepes (pH 7.4), 5% (v/v) glycerol, 1 mM EDTA (pH 8.0), 5 mM MgCl_2_, 0.1 mM TCEP and 0.1 mg/ml BSA.

##### Construction of DnaA_BA_ and its mutants

**Fig. 1:**
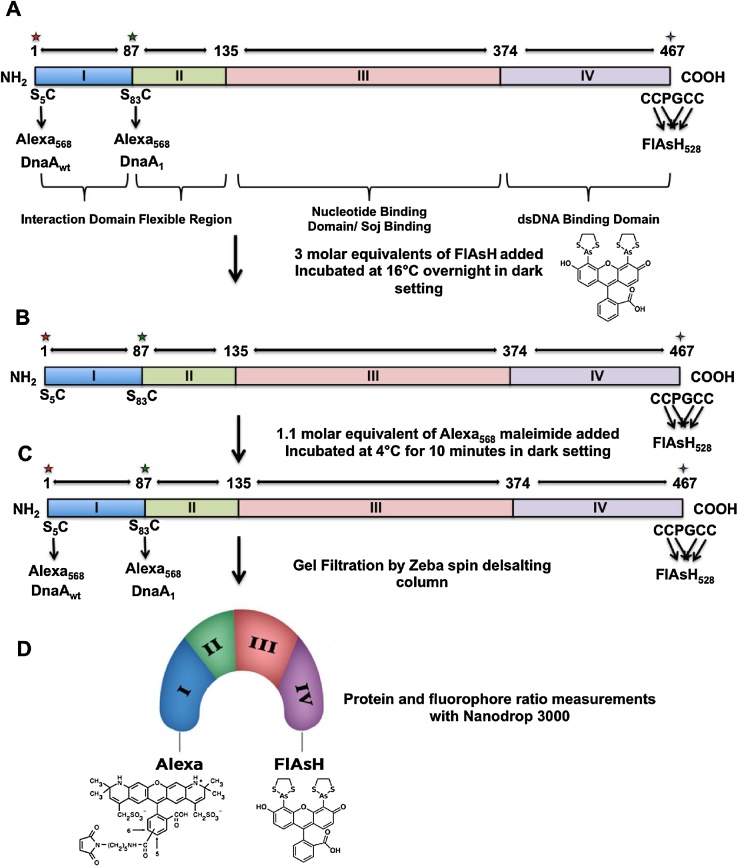
**Schematic representation of dual fluorophore labeling of DnaA protein**. (A) The DnaA protein was modified with a CCPGCC sequence at the C-terminus and single Cys motif at S5C or S83C, depending upon the construct. Two sites for Alexa_568_ maleimide labeling were chosen to view structural changes with substrate binding domains I–IV and domains II–IV. (B,C) The protein was then sequentially labeled with FlAsH fluorophore, followed by Alexa_568_ maleimide labeling in a dark setting. After site-specific labeling with FlAsH and Alexa_568_ maleimide fluorophores at the respective sites, gel filtration with a spin desalting column removed free fluorophores. (D) An illustration representing a model for DnaA_wt_ conformation double-labeled with Alexa_568_ and FlAsH. Protein concentrations were measured by a Bradford assay and Nanodrop was used to verify the recovery of double-labeled protein after gel filtration.

Absence of native cysteine (Cys) residues in DnaA_BA_ allowed us to easily introduce Cys residues at any desired location for site-specific labeling. We designed the DnaA_wt_ with a Cys residue at the N-terminus (S5C) and CCPGCCGG sequence (Cys4) at the C-terminus (Fig. 1) by PCR amplification with high-fidelity Q5 DNA polymerase (New England Biolab, Ipswich, MA) using the following oligonucleotides: 5′-TCTTCTCATATGGAAACATCTGTGATTTATGG-3′ (forward) and 5′-CTCCTCCTCGAGTCATTACTAGCCGCCACCGCAACAGCCAGGACAACACTTTAAAATATCGTTAATTTC-3′ (reverse). The DnaA_wt_ has the Cys residue and Cys4 sequence located in the designated sites to allow structural changes that occur in the full-length protein (Fig. 1A).

The DnaA_1_, DnaA_BA_ mutant with C83S and CCPGCCGGG at the C-terminus, was constructed by first PCR amplifying DnaA_BA_ using the following oligonucleotides: 5′-TCTTCTCATATGGAAAACATCTCTGATTTATGG-3′ (forward) and 5′-CTCCTCCTCGAGTCATTACTAGCCGCCACCGCAACAGCCAGGACAACACTTTAAAATATCGTTAATTTC-3′ (reverse), followed by *in vitro* mutagenesis (S83C) using Quik-Change Lightning Site-Directed Mutagenesis Kit (Fig. 1A) (Agilent, Santa Clara, CA). The DnaA_BA_ recombinant plasmids were fully sequenced in both directions by Eurofins MWG Operon LLC (Louisville, KY) and verified to be free of any fortuitous mutation(s) before expression and purification. The detailed procedure for expression and purification of proteins is discussed separately [7].

#### Labeling of proteins with FlAsH and Alexa_568_ maleimide

Purified DnaA_wt_ and DnaA_1_ proteins were tagged with the donor fluorophore, FlAsH, at the C-terminus and the acceptor fluorophore, Alexa_568_ maleimide, at S5C and S83C, respectively, as depicted in Fig. 1A. It should be noted that any buried Cys residue would prevent site-specific labeling by Alexa_568_ maleimide. In this study, we attempted to label a third DnaA construct with S183C for Alexa_568_ labeling. We were unsuccessful labeling this Cys residue presumably due to its intrinsically buried location, making it refractory to label with Alexa_568_ maleimide. Therefore, it is important that the Cys residue is located on the protein surface so that the fluorophores can easily crosslink to its sulfhydryl moiety.

The FlAsH and Alexa_568_ maleimide fluorophores work best in physiological conditions with a close to neutral pH of 7.5. Fluorescence labeling procedures were carried out in dark and using black polypropylene Eppendorf tubes (Thermo Scientific, Pittsburgh, PA). Purified recombinant protein (200 μg) was incubated at 16 °C overnight with 3 molar excess of FlAsH-EDT_2_ to crosslink with the C-terminus CCPGCC sequence (Fig. 1B). For overnight incubation of recombinant protein with FlAsH fluorophore, we used a PCR apparatus for controlled parameters of 16 °C for 12 h and then 4 °C until removed from PCR. Labeling the CCPGCC sequence with molar excess of FlAsH fluorophore first was done to avoid nonspecific labeling of Alexa_568_ maleimide to the four cysteines. FlAsH covalently attaches with all four Cys sulfhydryl groups in the Cys4 sequence at the C-terminus. Alexa_568_ maleimide (1.1 equivalent) was then added to the FlAsH-labeled protein at 4 °C and incubated for 10 min in a dark setting for single Cys labeling (Fig. 1C).

The double-labeled protein was immediately purified from unincorporated fluorophores using a 40 K, 0.5 ml Zeba desalting spin column (Thermo Scientific, Pittsburgh, PA) with labeling buffer as eluant. EDTA, mercaptoethanol, and DTT must be avoided as these have the potential to chelate the arsenics or quench FlAsH. For optimal labeling, the protein must be in the reduced form to avoid disulfide bridge formation and to allow fluorophore to cysteine-SH coupling reaction. TCEP (0.1 mM), with no free sulfhydryl group, was found to be a suitable reducing agent for site-specific labeling of proteins. Following the protocol, provided by the manufacturer, labeling buffer with TCEP was used to equilibrate the resin prior to using the spin-column to purify fluorophore-labeled proteins. The spin column was centrifuged at 1500 × *g* for two minutes on a bench-top microcentrifuge in a dark room. The purified double-labeled protein was aliquoted and stored in the same buffer at −80 °C. In addition, the spin column purification needed to be optimized for each protein before proceeding with fluorescence spectroscopy.

We determined DnaA protein concentration by Nanodrop 3000 measurement utilizing absorbance at 280 nm as well as Bradford protein estimation. Nanodrop was also used to determine fluorophore and protein concentrations and to confirm that the fluorophore and protein ratios were equivalent as well (should be ∼1:1 ratio for protein and FlAsH or Alexa_568_). Both measurements correlated well. We have evaluated donor/acceptor ratio by measuring fluorophore concentrations in double-labeled proteins by spectrophotometry at 528 nm (FlAsH with extinction coefficient of 70,000 cm^−1^ M^−1^) and 578 nm (Alexa568 with extinction coefficient of 91,300 cm^−1^ M^−1^). The acceptor to donor labeling ratio in the double-labeled proteins ranged between 0.95 and 1.05 in our preparations for FlAsH and Alexa_568_ double-labeled DnaA proteins. The fluorophore FlAsH to DnaA ratio was 1.0 ± 0.6 for analysis of FlAsH only single-labeled DnaA proteins.

#### Steady-state fluorescence spectroscopy and FRET measurements

We used FRET to measure changes in the distance between the fluorophores of the C- and N- terminus of the DnaA_wt_ and DnaA_1_ constructs under different conditions, enabling us to analyze the structural changes upon substrate binding (Fig. 1A). The construct DnaA_wt_, with labeling at both termini, allowed analysis of the structural changes occurring in the entire protein (domains I–IV). Like DnaA_wt_, DnaA_1_ was labeled with FlAsH at the C-terminus but with Alexa_568_ maleimide at Cys83. This enabled us to visualize structural changes occurring in domains II through IV, which are closer to domains III and IV, where ATP and DNA binding sites are located, respectively.

**Fig. 2:**
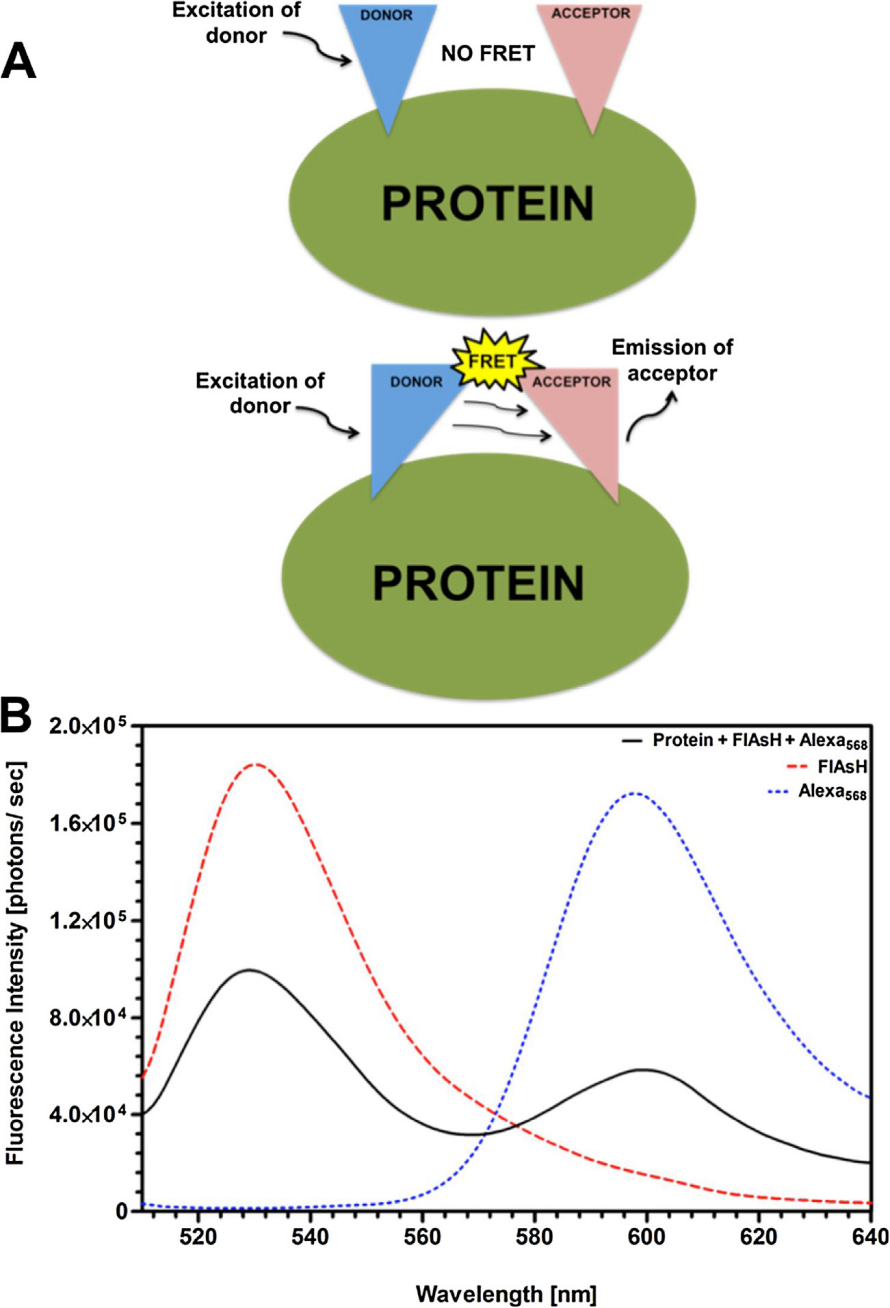
**Schematics of Energy Transfer in FRET**. (A) The donor and acceptor fluorophores must be in close proximity for energy transfer to occur. Fluorophores that are distally located do not undergo FRET, whereas fluorophores within 10 nm distance do show energy transfer upon excited donor fluorophore. This illustration depicts a donor and acceptor fluorophore labeling the same protein for intramolecular interaction to study conforamtional changes within a molecule. (B) The spectral overlap between the donor and acceptor peaks is a representation of FRET occuring within the double-labeled protein. The characteristic fluorescence spectrum of FlAsH () or Alexa_568_ () labeled proteins are shown. FRET spectrum of double-labeled protein demonstrated the energy transfer from FlAsH to Alexa_568_ (**^_____^**).

FRET requires the donor and acceptor fluorophores to be within ∼10 nm (100 angstrom) distance [4]. If the donor and acceptor fluorophores are located more than 10 nm apart on the protein, excitation of the donor will not transfer enough energy and excite the acceptor. However, if the donor and acceptor fluorophores are in close proximity within a protein, FRET will be detected for intramolecular structural analysis (Fig. 2A). The spectral overlap that occurs during FRET energy transfer from the donor fluorophore, FlAsH, to acceptor fluorophore, Alexa_568_, was visualized by fluorescence spectra of a single-labeled (FlAsH or Alexa_568_) and a double-labeled (FlAsH and Alexa_568_) protein as presented in Fig. 2B.

**Fig. 3:**
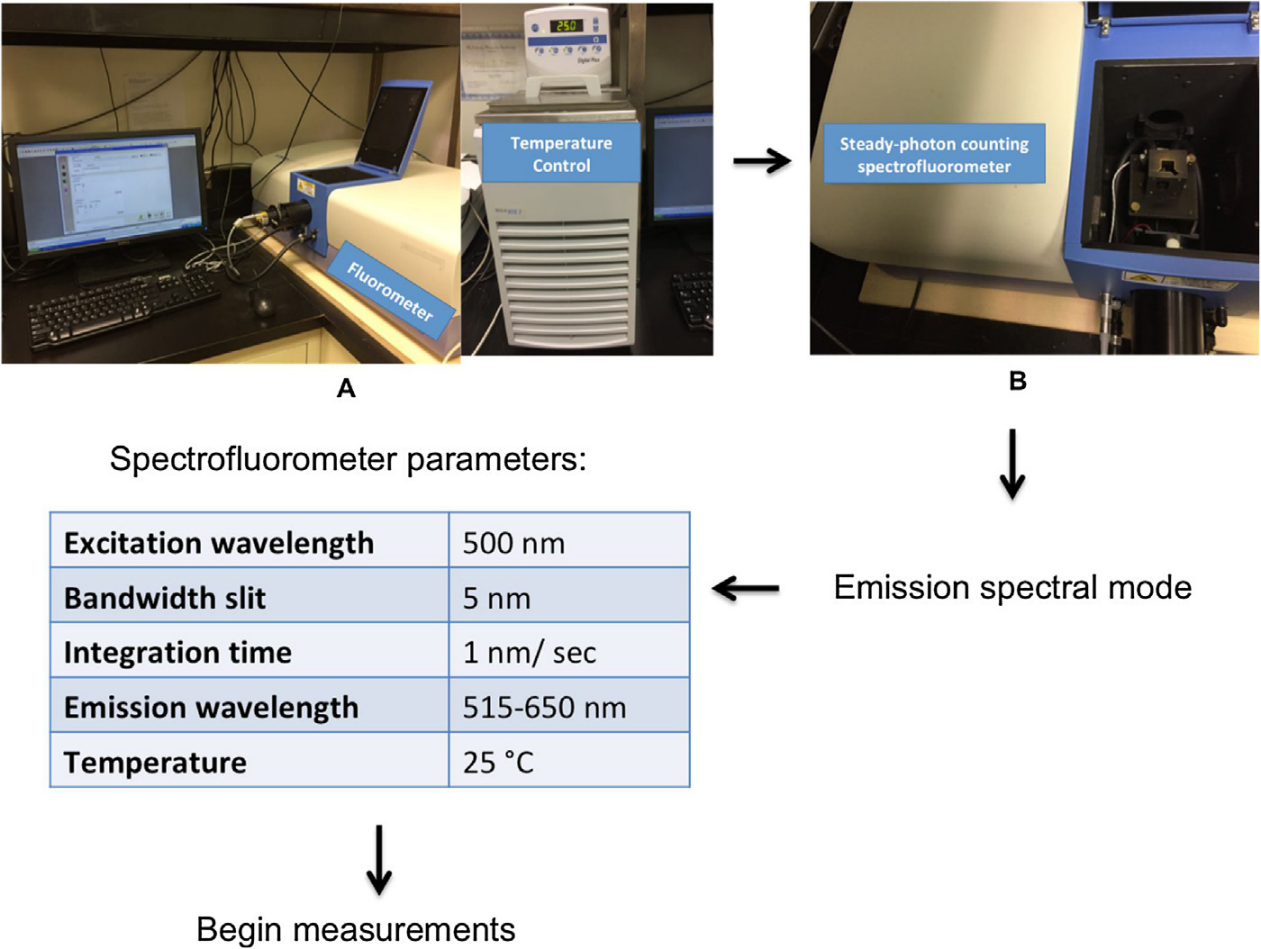
**Flow diagram of overall experimental workflow for steady-state photon counting spectrofluorometry**. (A) The Fluormax 4 spectrofluorometer by Horiba Scientific and FluorEssence software were used for acquiring the spectral emission data following the recommendations of the manufacturer. (B) A Quartz cuvette with the buffer containing labeled protein was inserted in the sample chamber. The optimal experiment settings were adjusted before the spectra was recorded as shown in the table. A blank reading with only anisotropy buffer was taken before enabling blank substraction for successive measurements.

All fluorescence measurements in our study were conducted using a steady-state photon counting spectrofluorometer. All experiments were performed in the dark at 25 °C as depicted in Fig. 3A. Fluorescence-labeled DnaA_x_ (0.2 μg) was added to 1 ml of anisotropy buffer, with a final concentration of 4 nM in a standard quartz cuvette (Fig. 3B). Horiba FluorEssence^TM^ software was used to collect spectral data and presentation, however a different steady-state photon counting spectrofluorometer with its respective software is suitable. Here, we will provide a general description of spectral data collection and analysis that can be used for any spectrofluorometer. Firstly, emission spectra is selected for experimentation. The optimal excitation wavelength, bandwidth, integration time, and wavelength range for the spectra is selected. In our case, the excitation wavelength was 500 nm with 5 nm slit width and integration time of 1 nm/s from 500 nm to 650 nm. The temperature controller is activated and set at 25 °C. A fluorescence scan was first taken with only the buffer as a blank to use for blank subtraction for subsequent measurements (Fig. 3).

Fluorescence spectra was recorded as triplicate scans for each run and automatically averaged. We repeated each run three times to obtain a total of 3 averaged scans for 3 accumulations or data for 9 total runs. For our study, fluorescence spectrum of each protein was measured independently as well as in the presence of 500 μM of nucleotide (ATP or ADP) and/or 100 nM of DnaA specific dsDNA. Protein was incubated with the substrates, such as nucleotides as well as DNA, for two minutes at 30 °C before measuring spectrum. Depending upon the study conducted by the researcher, the incubation time and temperature and ligand concentration can be adjusted.

#### Normalization of spectra

We used a free Fluortools program, specifically a/e-UV–vis-IR Spectral Software, for feasible raw fluorescence data normalization. This program can be downloaded to a Windows PC directly from http://www.fluortools.com/software/ae. Although we chose Fluortools, any other data normalizing software or manual normalization procedure can also be used. It is best to normalize the data in order to improve the integrity of the fluorescence emission data. Steady-state fluorescence spectra can change each time the experiment is conducted, however the pattern of the data should remain consistent for the experiment to be considered valid and reliable. Therefore, the raw data must be standardized in order to achieve accurate calculations.

To normalize our raw average spectrum, wavelength and fluorescence intensity data from fluorometer software were organized in Microsoft Excel spreadsheets. The data for double-labeled protein (DnaA in our studies) with or without the substrates (ATP +/− DNA and ADP +/− DNA) were selectively transferred from Microsoft Excel to the data-normalizing program. The data was normalized at the desired wavelength, which can be done manually or by the program chosen. The Spectral Software we chose allowed us to determine the optimum wavelength to use for normalization (http://www.fluortools.com/software/ae/documentation/edit-spectra/normalize).

Fluortools allows two methods of normalization: wavelength and area under the curve. We have tried using both to normalize; however with our study, normalization at wavelength appeared to work more easily and produced consistent spectra. The equation we used to normalize was [(intensity at wavelength/intensity at chosen wavelength to normalize) * multiplication factor]. The multiplication factor was chosen based upon trial and error to show the best normalized spectrum. After selection of this methodology, we used this algorithm for all of the data transformations through the remainder of our study [7]. Bulk FRET data can be corrected with either Fluortools or Microsoft Excel as discussed above.

#### Graphical representation

**Fig. 4:**
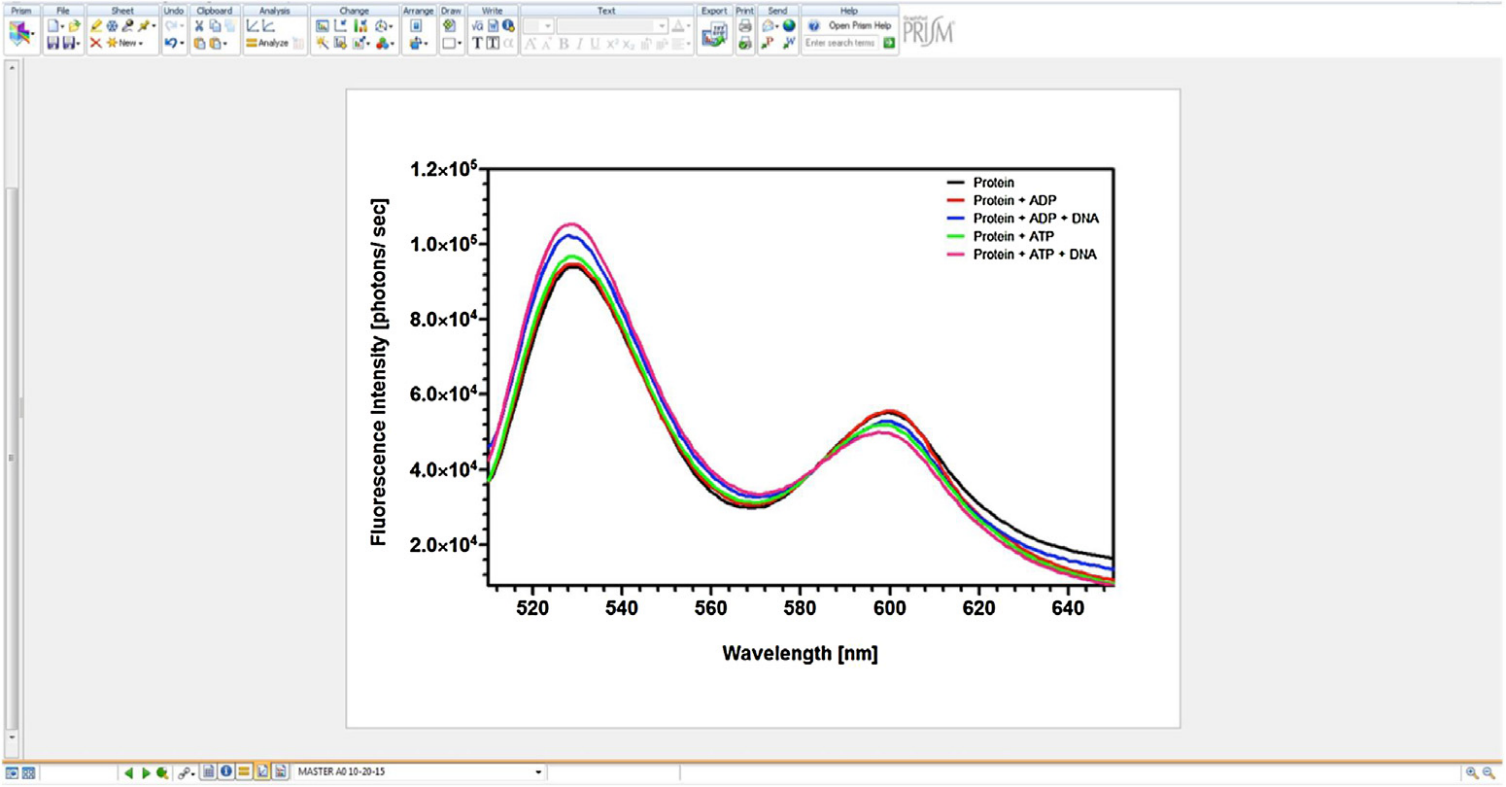
**Generating graphical representations of the normalized spectra using GraphPad Prism**. The normalized data from the Microsoft Excel spreadsheet was transferred to GraphPad Prism to create a line graph. A graph with wavelength as the x-axis and fluorescence intensity as the y-axis for the respective spectral data was produced. Fluorescence spectra for double-labeled DnaA protein and that with substrates depicting the structural changes are qualitatively shown by the spectra.

We used GraphPad Prism 5.0 software to generate graphical representations of the normalized spectra derived from Fluortools normalizations (https://www.graphpad.com/scientific-msoftware/prism/). The normalized data were transferred to GraphPad and the spectra were reconstructed, where the x-axis represented appropriate wavelength range (510–650 nm) and the y-axis represented the fluorescence intensities (Fig. 4). As shown in Fig. 4, the graphs were compiled in a master spreadsheet to plot the normalized spectra. Individual spectrum of the double-labeled protein with or without substrate(s) were then selected and presented to show the structural changes in the protein with changes in the FlAsH and Alexa_568_ emission peaks [7]. The spectra produced by GraphPad Prism were smoothened using the feature provided by the program.

#### Decomposition of spectra graphs by Gaussian fit

**Fig. 5:**
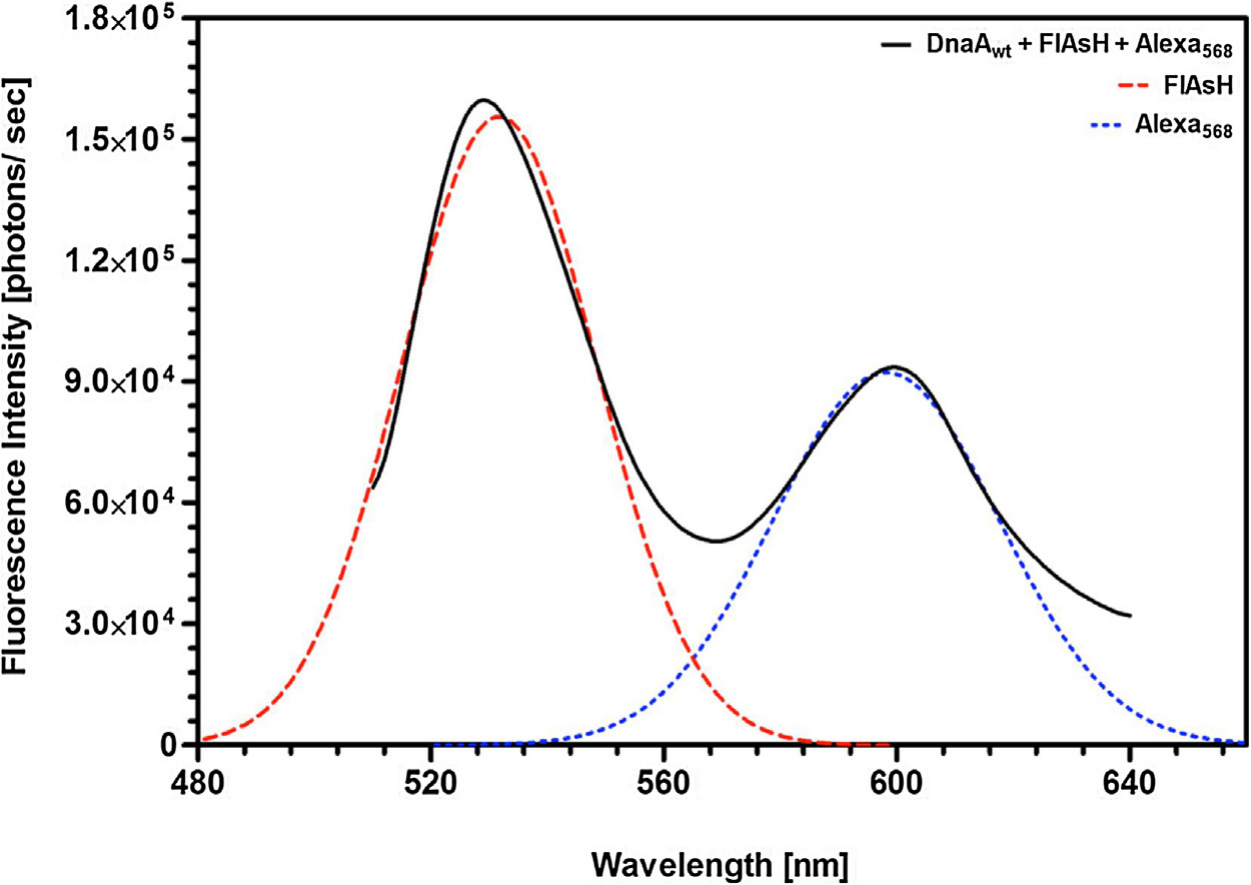
**Schematics of spectral fitting and peak resolution using a Gaussian function in the Fluortools software**. Decomposition of FRET spectrum was used to choose precise FlAsH and Alexa_568_ peaks for further quantitative analysis. FRET spectrum of the double-labeled (FlAsH and Alexa_568_) DnaA_wt_ (**^_____^**), contribution of FlAsH spectrum after decomposition (dashed red ) and Alexa_568_ spectrum after decomposition (dashed blue ) are displayed.

Gaussian fit by Fluortools was chosen to obtain precise individual donor FlAsH fluorophore and acceptor Alexa_568_ fluorophore emission peaks, denoted as I_D_ and I_A_ respectively, by decomposition. A Gaussian graph is composed by a quadratic equation and produces a symmetrical “bell” shaped curve. A detailed step-by-step procedure for modifying spectral bands to a Gaussian-shaped fit could be found from the free Fluortools software tool sets:

(http://www.fluortools.com/software/ae/documentation/tools/gaussianband). The program containing the Gaussian fit spectral band feature provided by Fluortools made the transition feasible rather than using complicated algorithms to decompose the spectra. The left and right boundaries of the FlAsH emission peak were selected and fit Gaussian first. The same procedure for FlAsH was repeated for Alexa_568_ emission peaks for a Gaussian shaped emission spectra (Fig. 5).

#### Calculating proximity ratio, efficiency of FRET and FlAsH-Alexa_568_ distance

**Fig. 6:**
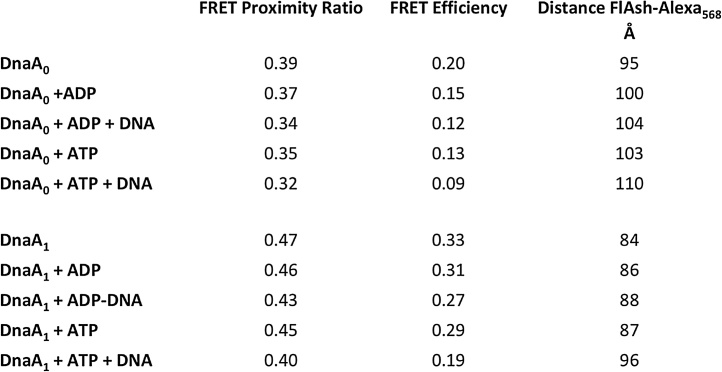
**Excel display of calculated proximity ratio, *E_FRET_,* and distances from FRET analysis**. These calculations were simplified by generating a Microsoft Excel spreadsheet. The proximity ratio was calculated using the *I_D_* and *I_A_* derived from the Gaussian fitted spectra (Eq. (1)) (Fig. 5). FRET efficiency (*E_FRET_*) was calculated with *F_D_* and *F_DA_* with the walvelength-normalized data (Eq. (2)). The FlAsH and Alexa_568_ distance (*r*) was derived from the experimental *E_FRET_* measure using a standard FRET equation (Eq. (3)) as described in Materials & Methods.

The Gaussian-shaped spectral bands gave us the intensities for the *I_D_* and *I_A_*, FlAsH and Alexa_568_ center peaks, respectively, and were used for determining proximity ratio, FRET efficiency and inter-fluorophore distance. This tool on Fluortools allowed us to shape the spectrum to be Gaussian shaped and isolate the total spectrum transition. In order to easily calculate proximity ratios for multiple conditions, we generated a Microsoft Excel spreadsheet with simple to use algebraic functions (Fig. 6). The proximity ratio was used as a relative measure of FRET to display qualitative structural/distance changes in a protein depending upon the FRET efficiency and the FlAsH-Alexa_568_ fluorescence quantum yield, shown in Eq. (1).

(1) P = *I*_A_/(*I*_A _+ *I*_D_)

The wavelength-normalized data was used to calculate efficiency of FRET (*E*_FRET_), which is the quantum yield of energy transfer shown in Eq. (2), and positively correlates to the FRET proximity ratio [1].

(2) *E*_FRET_ = 1 − (*F*_DA_/*F*_D_)

*F*_D_ and *F*_DA_ are FlAsH-only single-labeled protein and FlAsH-Alexa_568_ double-labeled protein, respectively. The *E*_FRET_ is a measure of the amount of energy that is transferred from the donor to acceptor and a function of the inter-fluorophore distance [3].By experimentally measuring *E* (or *E_FRET_*), the intramolecular structural changes were calculated by the distance between FlAsH and Alexa_568_ (*r*) determined by Eq. (3) [8,9],

(3) *r* = *R*_0_ [(1 − E)/E]^1/6^

*R_0_*, a constant of donor-acceptor distance where 50% of the energy is transferred, can be calculated using Eq. (4),

(4) *R*_0 _= (9.765 × 10^3^) (j (λ) κ^2^ Φ_D_*n*^−4^)^1/6^

*R*_0_ for this FRET pair has been established earlier to be 75 Å as the standardized value for the FlAsH-Alexa_568_ donor-acceptor pair by Granier et al. [2]. Since *R_0_* is a constant that is determined based upon which donor and acceptor fluorophores are used, in our case FlAsH-Alexa_568_, we continued with the predefined value for our FRET analysis. A Microsoft Excel spreadsheet with each of these formulas was created to easily calculate the values of *r* as well (Fig. 6).

**Fig. 7:**
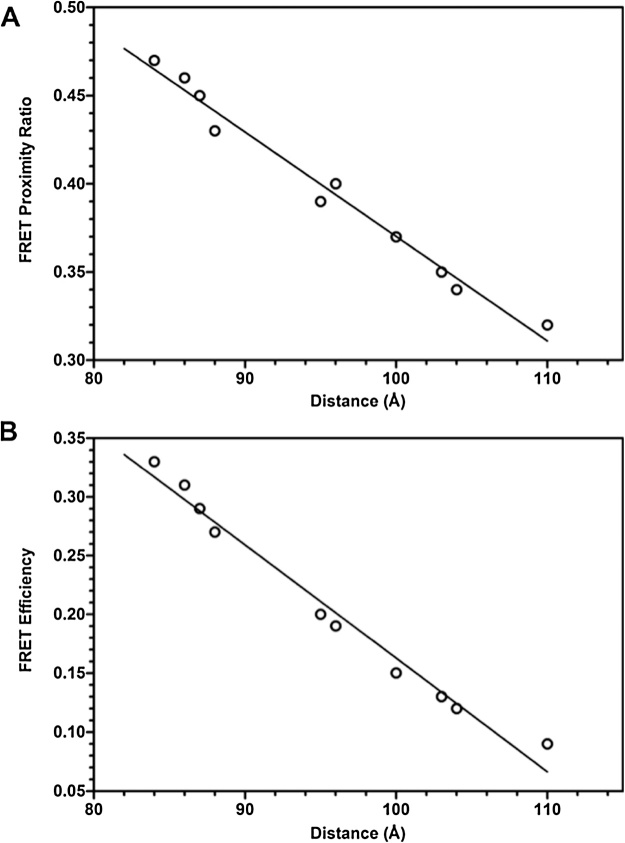
**Correlation between the FlAsH-Alexa_568_ distance (*r*) and FRET proximity ratios as well as FlAsH-Alexa_568_ distance (*r*) and FRET efficiency**. (A) As the distance between the fluorophores increases, FRET proximity ratio (Eq. (1)) decreases due to the reduction of the energy transfer efficiency. Proximity ratio is a qualitative measure for structural changes in a protein. (B) As the distance between fluorophores increases from structural changes, FRET efficiency (Eq. (2)) decreases when the donor fluorophore inefficiently transfers energy to acceptor fluorophore. FRET efficiency is a quantitative measure of structural changes and positively correlates to FRET proximity ratio. The distance (Eq. (3)) shows the closer or farther movement between the fluorophores, quantitatively showing the structural changes the protein is undergoing. The linear regression line in the plot demonstrates a putative linear relationship between these two FRET-derived functions and fluorophore distance changes in our study, demonstrating a clear conformational change undergone by the protein.

When calculating and creating graphical representations of our data, we noticed a linear correlation between the distance and the proximity ratio as shown in Fig. 7A. This pattern between the inter-fluorophore distance and FRET proximity ratio made logical sense between the relationships, as mentioned earlier. As the distance between the fluorophores (*r*) increases, less energy transfer occurs and consequently, the FRET proximity ratio decreases (Fig. 7A). The linear correlation graph was derived from the FRET-based structural study of DnaA protein [7]. An identical linear correlation between the distance and FRET efficiency was observed as well (Fig. 7B). Fig. 7B shows that as the fluorophores move apart due to conformational changes by the protein, the efficiency of energy transfer is reduced depicted by FRET efficiency. These plots validate the use of proximity ratio as a qualitative predictor and FRET efficiency as a quantitative predictor of the protein structural change.
